# Repurposing Ziyuglycoside II Against Colorectal Cancer *via* Orchestrating Apoptosis and Autophagy

**DOI:** 10.3389/fphar.2020.576547

**Published:** 2020-09-18

**Authors:** Can Bai, Zhe Zhang, Li Zhou, Huan-Yu Zhang, Yan Chen, Yong Tang

**Affiliations:** ^1^ Acupuncture and Tuina School, Chengdu University of Traditional Chinese Medicine, Chengdu, China; ^2^ State Key Laboratory of Biotherapy and Cancer Center, West China Hospital, and West China School of Basic Medical Sciences & Forensic Medicine, Sichuan University, and Collaborative Innovation Center for Biotherapy, Chengdu, China; ^3^ Key Laboratory of Tropical Diseases and Translational Medicine of Ministry of Education & Department of Neurology, The Affiliated Hospital of Hainan Medical College, Haikou, China

**Keywords:** Ziyuglycoside II, cancer therapy, autophagy, colorectal cancer, Akt

## Abstract

Effective chemotherapy drugs for colorectal cancer remain a challenge. In this research, Ziyuglycoside II (Ziyu II), exhibits considerable antitumor activity against CRC cells both *in vitro* and *in vivo*. The results showed that Ziyu II induced apoptosis through the accumulation of reactive oxygen species (ROS), which was necessary for Ziyu II to inhibit colorectal cancer cells. Intriguingly, The treatment of Ziyu II triggered complete autophagic flux in CRC cells. Inhibition of autophagy partially reversed Ziyu II-induced growth inhibition, demonstrating a cytotoxic role of autophagy in response to Ziyu II-treated. Mechanism indicated that Ziyu II-induced autophagy by inhibiting Akt/mTOR pathway. Akt reactivation partially reduced Ziyu II-induced LC3-II turnover and LC3 puncta accumulation. Especially, Ziyu II improves the sensitivity of 5-fluorouracil which is the first-line chemotherapy drug in colorectal cancer cells. This research provides novel insight into the molecular mechanism of Ziyu II’s anti-proliferation, including apoptosis and autophagy, and lays a foundation for the potential application of Ziyu II in clinical CRC treatment.

## Introduction

Colorectal cancer (CRC) has been associated as the most generally diagnosed cancers worldwide, with the number of new cases relinquished 1.85 million in 2018, accounting for 9.2% of all cancer-related deaths (Siegel et al., 2017; Bray et al., 2018). Meanwhile, the rate of CRC is growing fast with insufficient therapeutic options. Screening for colorectal cancer remains the most significant and cost-effective maneuvering to diminish the incidence rate and mortality of the illness, but it still lacks the screening programs in the developing countries (Kuipers et al., 2015; Onyoh et al., 2019). Besides, surgery, radiotherapy, and adjuvant chemotherapy have greatly improved the survival rate of colorectal cancer, but there are some obstacles in the application of chemotherapy, such as lack of selectivity, insufficient drug concentrations in tumor tissues, the emergence of drug-resistant cancer cells, and inevitable systemic toxicity (Geng et al., 2017). Hence, novel agents with rare or no by-effects are urgently needed to improve the prognosis of CRC patients.

Autophagy has recently introduced significant attention due to its important roles in human diseases and gained a Nobel Prize for physiology or medicine in 2016 (Mizushima, 2018). Autophagy generally includes three types that are macro-autophagy, micro-autophagy, and chaperone-mediated autophagy (CMA) (Lin and Baehrecke, 2015; Ruocco et al., 2016). Macro-autophagy (hereafter mentioned to as autophagy) is an evolutionarily conserved physiological process by which damaged organelles and macromolecules are degraded and recycled for cell survival and proliferation under physiologic conditions, such as accumulation of reactive oxygen species (ROS) and energy limiting (Mejlvang et al., 2018; Mizushima, 2018). Meanwhile, it also occurs frequently during tumorigenesis and cancer chemotherapy (Poillet-Perez et al., 2018; Chen et al., 2019), even related to cancer drug resistance (Sun et al., 2017). Owing to its “self-digest” function, the role of autophagy in cancer is complicated and context-dependent (Kimmelman and White, 2017). A growing number of reports indicate that targeted autophagy process has been considered as a novel therapeutic approach (Lei et al., 2017; Sun et al., 2019). Therefore, the development of novel autophagy regulators has redesigned a way of cancer treatment in recent years.

**Figure 1:**
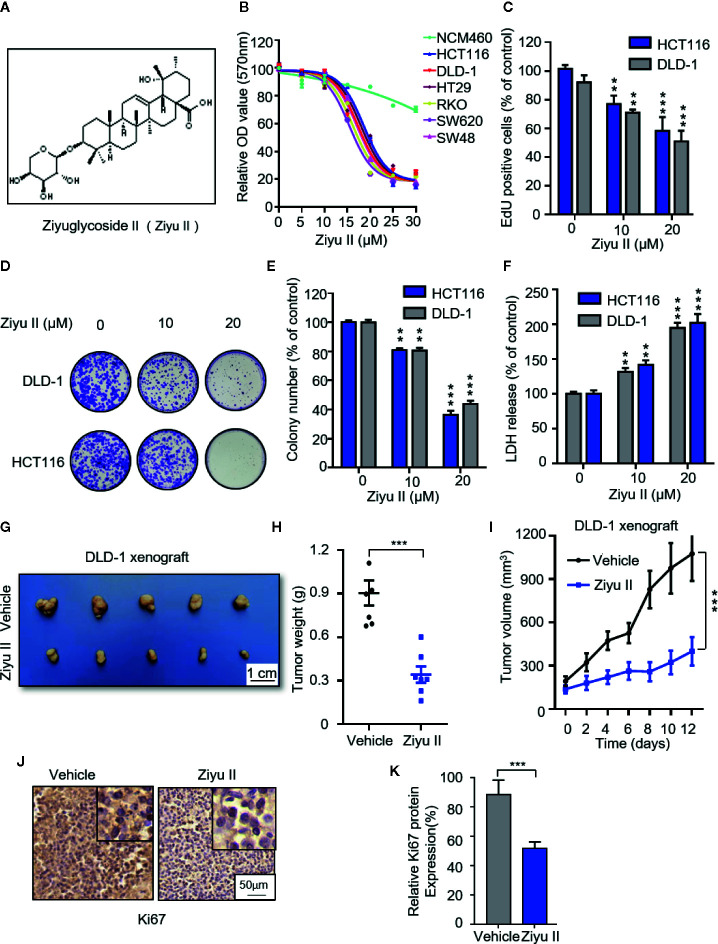
Ziyuglycoside II inhibits colorectal cancer cells proliferation both *in vitro* and *in vivo.* **(A)**. Chemical structure of Ziyu II. MTT assay of colorectal cancer lines incubated with the designated concentrations of Ziyu II for 24 h **(B)**. EdU incorporation **(C)**, LDH release assay in cells incubated as in **(F)**. Colony formation assay of DLD-1 and HCT116 cells intervented with the designated concentrations of Ziyu II for 2 weeks. Characteristic images **(D)** and quantification of colonies **(E)** were shown. G–J, NOD-SCID mice were injected with DLD-1 cells and intervented with Ziyu II or vehicle. Tumor volumes were measured at designated time points **(I)**. Photograph of dissected tumors derived from control or Ziyu II-treated mice **(G)**. Tumor weights at time of sacriﬁce **(H)**.The expression of Ki67 was detected by IHC in tumor xenografts **(J)**, and relative immunohistochemical scores were shown **(K)** Scale bar, 50 μm. All data are means ± SD. **p < 0.01; ***p < 0.001.

Drug repurposing has recently emerged as an alternative approach to accelerate the development for cancer treatment. Repurposing the large arsenal of non-anticancer drugs holds promise to achieve a rapid clinical practice at a lower cost than *de novo* drug development (Corsello et al., 2017). In the past decades, traditional Chinese medicine (TCM) has drawn growing attention as special drug pool for drug repurposing in the cancer management. Ziyuglycoside II (3β-3-α-L-arabinopyranosyloxy-19-hydroxyurs-12-en-28-oic acid) (**Figure 1A**) is one of the main active compounds of *Sanguisorba officinalis L*, which is spread in the north temperate zone of Asia and Europe, especially in China. And it has extensive clinical applications including antibiosis, anti-inflammation, anti-oxidation, and anti-cancer. Previous studies have identified Ziyu II as an effective anti-tumor agent that inhibits the proliferation of the cancer cells by triggering apoptosis and inducing cell cycle arrest in diverse cancers, including breast cancer and gastric cancer (Wang et al., 2011; Zhu A. K. et al., 2013; Zhu et al., 2014; Dou et al., 2016). Nevertheless, the specific mechanism underlying the anticancer effect of Ziyu II remains to be further defined.

In this study, we demonstrated that Ziyu II induces growth inhibition and obvious cell death of CRC cells by triggering autophagy and apoptosis both *in vitro *and* in vivo*. Notably, Ziyu II promotes complete autophagic flux which results from the repression of the Akt/mTOR signaling pathway, leading to growth inhibition of CRC cells. Moreover, Ziyu II synergistically suppresses CRC cell growth with the first-line chemotherapeutic drugs 5-fluorouracil. Together, our findings elucidated the mechanisms of Ziyu II-induced growth inhibition of CRC cell by orchestrating both apoptosis and autophagy, which may pave the way for the use of Ziyu II in-clinic treatment of CRC.

## Methods

### The Cell Proliferation Inhibition Test

The MTT assay was used to detect the cell growth rate. Briefly, cells were plated in 96-well plates (4 x 10^3^ per well) and received different concentrations of drug treatment.

The detailed procedures have been described by Dou et al. (2016). The colony formation assay, cells were cultured in 24-well plates (800 cells/well) and treated with different treatments. After 2 weeks, the 4% paraformaldehyde was adopted for fixation and crystal violet was used for dyeing. The visible colonies were captured by Molecular Imager Gel Do XR + System (Bio-Rad, Hercules, CA, USA) and calculated applying ImageJ software (National Institutes of Health, Bethesda, MD, USA). The detailed procedures have been previously described by Wang et al. (2011). The 5-ethynyl-20-deoxyuridine (EdU) labeling analysis was carried out in 24-well plates (2 x 10^4^ cells) using the EdU cell Proliferation Assay Kit (Ribobio, Guangzhou, China). After different concentrations of drug treatment, 10 μmol/L EdU was added to the cells, and the cells were incubated for 12 h at 37°C. Cells were fixed with 4% paraformaldehyde. Then DAPI was utilized for nuclear staining and fluorescence microscopy was applied for imaging.

### Determination of Lactate Dehydrogenase Release

Lactate dehydrogenase (LDH) test kit (Beyotime Biotechnology, Nanjing, China) was used to assess the cytotoxicity of Ziyu II. Cells were cultured in 96-well plates (6 x 10^3^ cells/well). After treatment with various concentrations of Ziyu II for 24 h, cell culture supernatant was transferred to the new 96-well plate for LDH analysis.

### Measurement of Intracellular ROS Levels

The level of reactive oxygen species was quantified according to the scheme of active oxygen analysis kit (China beiotim biotechnology company). Cells were incubated in 6-well plates (3 x 10^5^ per well) treated with designated concentrations of Ziyu II for 24 h and dyed with Muse™ Oxidative Stress reagent at 37°C for 30 min. Cells were then collected for ROS analysis using the FACSCalibur flow cytometer (BD Biosciences, San Jose, CA, USA).

### Flow Cytometry

According to the manufacturer’s instructions, the apoptotic cell ratio was measured using an annexin V–FITC/propidium iodide (PI) Detection Kit (KGA108; KeyGen Biotech, Nanjing, China). Cells were collected, washed twice with PBS, and then resuspended in 500 μl binding buffer. After 5 μl annexin V—FITC and 2 μl PI were added to the cell suspension, at least 20,000 living cells were analyzed on fascalibur flow cytometry (BD Biosciences, San Jose, CA, USA). Data were analyzed using FlowJo software (FlowJo, Ashland, OR, USA).

### Plasmids

The human AKT (NM_001014431.1) coding region with GFP-Tag was cloned using PCR and was ligated into the pEGFP-N1 vector. The original PCR primers for AKT cds sequence were as follows: Sense primer: 5’ CCG GAA TTC ATG AGC GAC GTG GCT ATT 3’; Anti-sense primer: 5’CGC GGA TCC CCG GCC GTG CCG CTG GCC GA 3’. The constitutively active form of Akt (CA-Akt, myrAkt delta4-129) was bought from Addgene. Flag-Ubiquitin was a kind gift from Professor Changan Jiang (Sichuan University, China). According to the manufacturer’s instructions, the plasmid was transfected into the cells using liposome 3000.

### Animal Model

All animal experiments were approved by the Institutional Animal Care and Treatment Committee of Sichuan University. Female BALB/c nude mice, aged 5 weeks, were purchased from HFK Bioscience Co., Ltd (Beijing, China). The animals were placed under standard conditions. In the subcutaneous xenograft model, the flanks of mice subcutaneously were injected with DLD cells (1 x 10^7^ cells/mouse) suspended by PBS. When the tumor volume reached ~50 mm^3^, mice were randomly divided into two groups. The mice were administered with 0.1 ml vehicle (10% ricinus oil, 5% DMSO, 10% ethanol, 75% physiologic saline) or Ziyuglycoside II (50 mg/kg/d) by gavage. The tumor volumes were measured every other day and evaluated according to the following formula: tumor volume(mm^3^) = (length x width^2^)/2. 4 weeks post treatment, the tumors were harvested when the mice were euthanized.

### Immunohistochemical Analysis

Immunohistochemical analysis was performed as described previously (Wang et al., 2011). All samples were observed under a Leica DM 2000 microscope. The percentage of positive cell area (a) was multiplied by the intensity of immunostaining (B: 0, negative; 1, weak positive; 2, positive; 3, strong positive). The calculation of A × B was taken as the final score of each slide.

### Immunoblot

Cells were lysed with RIPA buffer (150 mmol/L Tris-HCl pH 7.0, 150 mmol/L NaCl, 1% NP-40, 1% Sodium deoxycholate, 0.1% SDS) containing with protease inhibitor cocktail (Sigma, p8340) for 30 min at 4°C. The Bio-Rad protein assay quantified the lysates. The protein samples were then subjected to SDS-PAGE and probed with the indicated antibodies to visualize the protein levels using a chemiScope 6000 Touch (Clinx, Shanghai, China).

### Statistical Analysis

All statistical analysis was performed using GraphPad Prism Software version 6.0. All experiments were performed three or more times independently. For two-group comparisons, the student’s two-tailed t-test was used. For multiple group comparisons, one-way ANOVA analysis was used. *P* < 0.05 was defined as statistically significant.

### Materials

#### Cell Culture

Human colorectal cancer cell lines (HCT116, HT29, DLD-1, SW620, and SW480) and the human normal colonic epithelial cell line NCM460 were obtained from the ATCC (Manassas, VA, USA) and cultured in DMEM (Thermo Fisher Scientific, Waltham, MA, USA) supplemented with 10% fetal bovine serum (Thermo Fisher Scientific), 100 U/ml penicillin (Millipore Sigma, Burlington, MA, USA), and 100 U/ml streptomycin (Millipore Sigma) at 37°C in 5% CO_2_.

### Antibodies and Reagents

Antibodies were purchased from Cell Signaling Technology (ATG5 12994S, ATG7 8558S, Akt 4685, p-Akt 4060, mTOR 2972, p-mTOR 2971, p70S6K 9202, p-p70S6K 9208, 4E-BP1 9452, p-4E-BP1 9451), Abcam (PARP ab74290, cleaved-PARP ab32064, Ki67 ab66155, Santa Cruz Biotechnology (β-actin sc-1616, horseradish peroxidase-conjugated anti-rabbit secondary antibody sc-2004, horseradish peroxidase-conjugated anti-mouse secondary antibody sc-2005), Thermo Fisher Scientific (goat anti-mouse Alexa Fluor 488, goat anti-rabbit Alexa Fluor 488, goat anti-mouse Alexa Fluor 594, goat anti-rabbit Alexa Fluor 594), and Novus (LC3 NB100-2220). DAPI (62248) and Lipofectamine 3000 (L3000015) were purchased from Thermo Fisher Scientific. Unless otherwise indicated, all commercial chemicals were purchased from Med-Chem Express (Monmouth Junction, NJ, USA).

## Results

### Ziyuglycoside II Represses Colorectal Cancer Cells Growth Both *In Vitro* and *In Vivo*

To examine whether Ziyu II exhibits an antitumor effect against CRC, CRC cell lines (HCT116, DLD-1, HT29, SW48, SW620, RKO) and noncancerous colorectal cell line (NCM460) were treated with different dose of Ziyu II. MTT assay showed that Ziyu II markedly inhibited the growth of CRC cells in a dose-dependent manner, whereas the IC50 value in NCM460 cells was much higher than those in CRC cells (**Figure 1B**). As the decrease of colony formation and EdU incorporation show, the proliferation of colorectal cancer cells decreased significantly after Ziyu II treatment (**Figures 1C–E**). Then we performed LDH release assay and found that Ziyu II damaged the integrity of the plasma membrane (**Figure 1F**). In summary, these results demonstrated that Ziyu II inhibits the growth of CRC cells *in vitro*.

To further ascertain the antitumor function of Ziyu II on CRC growth *in* *vivo*, and by subcutaneously injecting DLD-1 cells into nude mice, we generated a CRC xenograft model. As shown in **Figures 1G, H**, Ziyu II treatment markedly decreased the size and the weight of xenografts when compared with the control group. Besides, the growth rate of xenograft treated with Ziyu II was slower than that of the placebo group (**Figure 1I**). Moreover, most of Ziyu II-treated tumors displayed reduced Ki67 staining (**Figures 1J, K**). Furthermore, we noticed that Ziyu II treatment had no significant effect on the pathological features of major organs, suggesting that Ziyu II has no obvious toxic or adverse effect on mice (**Figure S1**). In brief, these data indicated which Ziyu II represses the growth of CRC cells both *in* *vitro* and *in* *vivo*.

### Ziyuglycoside II Induces Apoptosis in Colorectal Cancer Cells Both *In* *Vitro* and *In* *Vivo*

**Figure 2:**
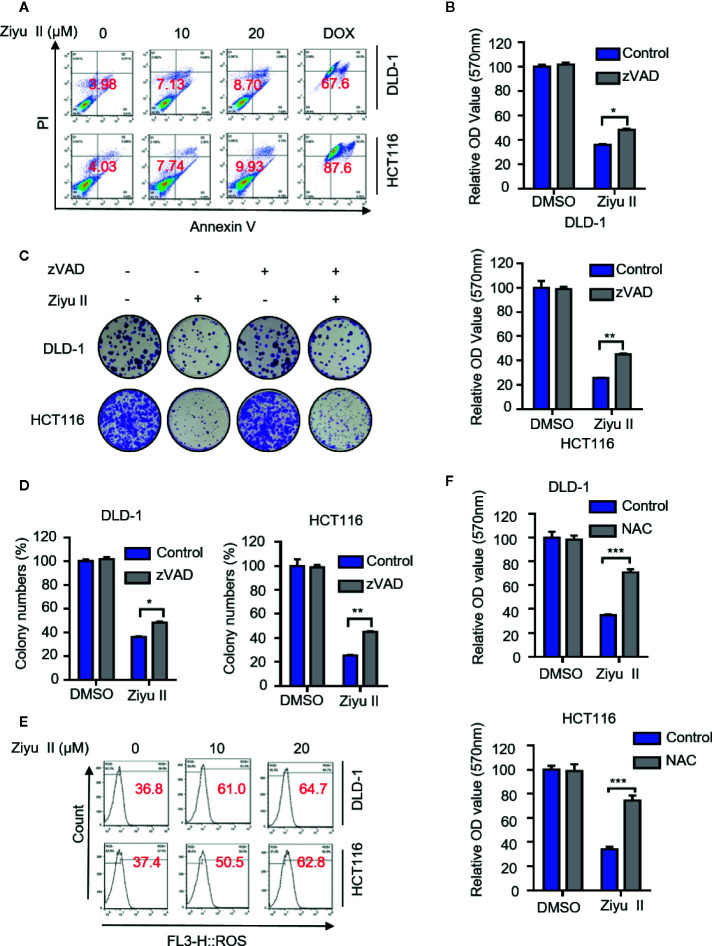
Ziyuglycoside II induces apoptosis in colorectal cancer cells *in vitro.* DLD-1 and HCT116 cells were subjected to Ziyu II Dox 10uM for 24 h, and flow cytometric analysis of apoptosis **(A)**. **(B)** MTT assay of DLD-1 and HCT116 cells indicated with the presence or absence of Ziyu II, and in combination with or without ZVAD for 24 h. Colony formation assay of DLD-1 and HCT116 cells indicated with the presence or absence of Ziyu II, and in combination with or without ZVAD for 2 weeks. The characteristic images **(C)** and count of colonies **(D)** were shown. **(E)** Flow cytometric analysis of ROS in DLD-1 and HCT116 cells treated with the indicated concentration of Ziyu II for 24 h. **(F)**. MTT assay of DLD-1 and HCT116 cells indicated with NAC for 24 h. All data are means ± SD. *p < 0.05; **p < 0.01; ***p < 0.001.

Apoptosis is a major form of cell death induced by chemotherapy drugs and has been widely documented. Previous studies suggested that Ziyu II exerted antitumor activity by inducing apoptosis in human breast cancer and gastric cancer cells (Zhu X. et al., 2013; Zhu A. K. et al., 2013). To get more insights into the mechanism of Ziyu II-induced cell death, we detected whether Ziyu II induced apoptosis in CRC cells. Firstly, Ziyu II treatment resulted in increased levels of cleaved-caspase3, a prototypical caspase that becomes activated during apoptosis (Letai, 2017).and cleaved-PARP and decreased expression of anti-apoptotic protein Bcl2 (**Figure S2A**). Consistently, the flow cytometry analysis exerted remarkable apoptotic effect in Ziyu II treated CRC cells (**Figure 2A**). To further validate Ziyu II induced apoptosis, CRC cells were treated with Ziyu II combination with the apoptosis inhibitor-zVAD. As shown in **Figure S2B**, combinational treatment resulted in reduced levels of cleaved-PARP. Consistently, the pro-apoptotic effect of Ziyu II was partially rescued under the combinational treatment, as evidenced by MTT assay and colony formation (**Figures 2B–D**). As the accumulation of reactive oxygen species (ROS) plays an essential function in the induction of apoptosis, we detected whether Ziyu II treatment promoted ROS accumulation. The flow cytometry analysis showed that Ziyu II markedly raised the level of ROS in a dose-dependent manner in CRC cells (**Figure 2E**). What’s more, MTT assay showed that N-acetylcysteine (NAC, a ROS scavenger) significantly reversed Ziyu II-induced growth inhibition (**Figure 2F**). In brief, these results demonstrate the pro-apoptotic effect of Ziyu II in CRC cells* in vitro*.

To assess the pro-apoptotic effect of Ziyu II on CRC cells *in vivo*, xenografts were stained for cleaved-caspase3. As shown in Fig. S2C and D, Ziyu II-treated xenografts displayed stronger cleaved-caspase3 staining than that of placebo-treated xenografts. In brief, these data represent that apoptosis is partaken in Ziyu II against CRC cells both *in* *vitro* and *in* *vivo*.

### Ziyuglycoside II Provokes Autophagosome Formation in Colorectal Cancer Cells

**Figure 3:**
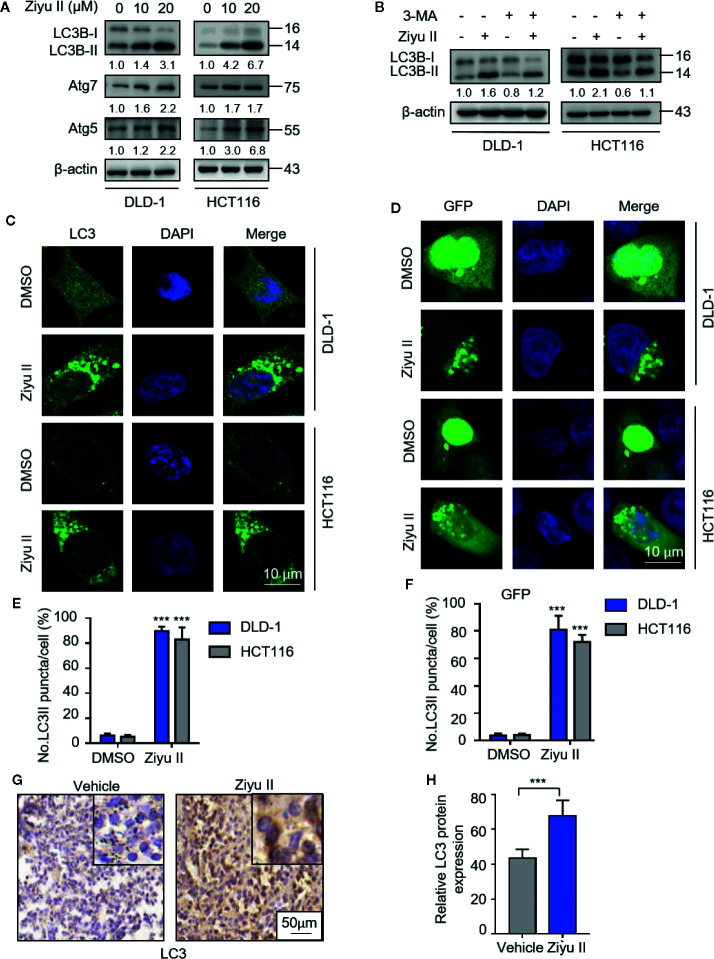
Ziyuglycoside II induces autophagy in colorectal cancer cells both *in vitro* and *in vivo.* **(A)** The protein of LC3, Atg7, and ATG5 of DLD-1 and HCT116 cells were detected by Western blot after 24 h of treatment with Ziyu II. **(B)** Immunoblotting of LC3 in DLD-1 and HCT116 cells indicated the presence or absence of Ziyu II, and in combination with or without 3 MA for 24 h **(C)** Formation of endogenous LC3 puncta in cells exposed to DMSO or Ziyu II for 24 h and **(E)** total number of endogenous LC3 puncta per cell. **(D)**, the formation of exogenous GFP-LC3 puncta in cells treated with DMSO or Ziyu II for 24 h and **(F)** the total number of exogenous LC3 puncta per cell. Scale bars: 10 μm. LC3 expression in orthotopic xenografts was examined by IHC. Representative images were provided as indicated **(G)**, and relative immunohistochemical scores were shown **(H)**. Scale bar, 50 μm. All data are means ± SD. **p < 0.001.

As pieces of evidence highlighted the important role of drug-induced autophagy in cancer treatment (Cui et al., 2018; Poillet-Perez et al., 2018; Deng et al., 2019), we examined whether autophagy is involved in Ziyu II-induced CRC cell death. We first detected the levels of autophagy-related proteins in Ziyu II-treated CRC cells and found that Ziyu II treatment promoted the turnover of LC3-I to lipidated LC3-II in a dose-dependent manner in CRC cells (**Figure 3A**). Also, we evaluated the expression levels of ATG5 and ATG7, two autophagy-related proteins, which are notably increased in a dose-dependent manner after Ziyu II treatment (**Figure 3A**). When combined with 3-MA, an inhibitor of class III PI3K, the elevated LC3-II levels were prominently inhibited in Ziyu II-treated CRC cells, suggesting that Ziyu II induces autophagy initiation (**Figure 3B**). This was further confirmed by siRNA-mediated *ATG5* silencing, as evidenced by decreased LC3 puncta in the combinatorial treatment group (**Figures S3A–E**). Moreover, The autophagic vacuoles (Lee et al., 2017) which were represented by the endogenous LC3 and exogenous GFP-LC3 puncta were remarkably increased in Ziyu II-treated CRC cells(**Figures 3C–F**). Besides, The LC3 staining of the xenografts treated with Ziyu II displayed stronger than that of the control group (**Figures 3G, H**). Taken together, these results show that Ziyu II promotes the initiation process of autophagy in CRC cells.

### Ziyuglycoside II Promotes Autophagic Flux in Colorectal Cancer Cells

**Figure 4:**
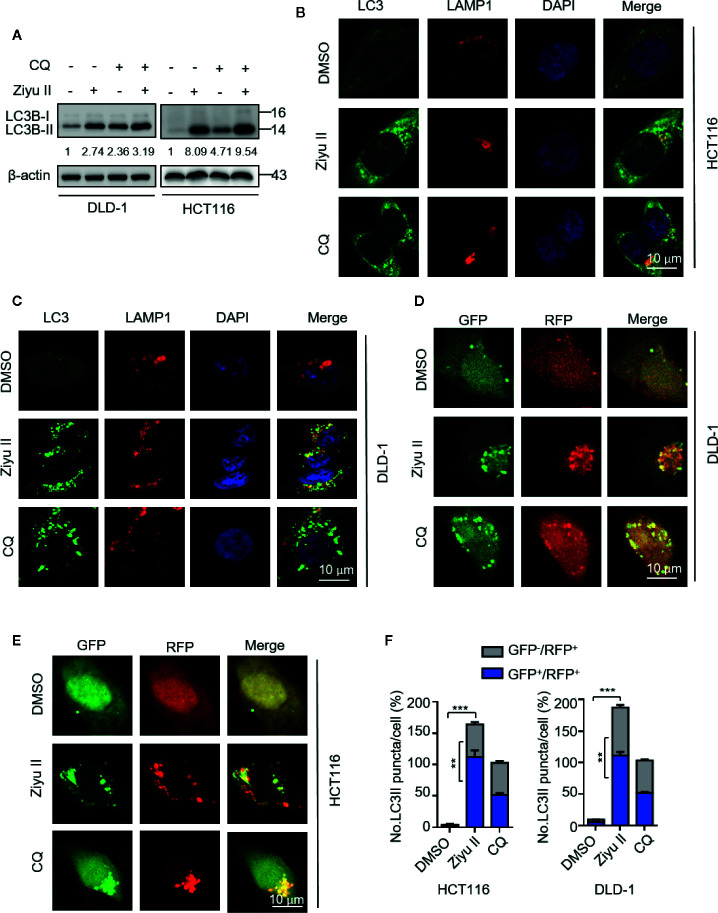
Ziyuglycoside II promotes autophagy flux in colorectal cancer cells. **(A)** Immunoblotting of LC3 in cells treated with or without the Ziyu II for 24 h. **(B, C)** The colocalization of endogenous LC3 with LAMP1 was detected by immunoﬂuorescent analysis after treatment of Ziyu II and CQ (10 mM) for 24 h, respectively. **(D–F)** DLD-1 and HCT116 cells were transiently transfected with an RFP-GFP tandem ﬂuorescent-tagged LC3 (RFP-GFP-LC3) and indicated with Ziyu II and 10 mmol/L chloroquine (CQ) for 24 h, respectively. The formation of autophagosome (RFP-positive; GFP-positive) and autophagolysosome (RFP-positive; GFP-negative) were detected and quantified by ImageJ. Scale bars: 10 μm. All data are means ± SD. **p < 0.01, ***p < 0.001.

The elevated levels of LC3-II may be associated with either autophagy triggering or obstructive of autophagic flux. Thus, we determined whether Ziyu II-induced complete autophagic flux. First, we detected the protein levels of LC3-II under the combinatorial treatment of Ziyu II with chloroquine (CQ), an autolysosome inhibitor. As expected, combinatorial treatment resulted in enhanced accumulation of LC3-II (**Figure 4A**). Moreover, we also identified the colocalization of LC3 (which is the autophagosome marker) with LAMP1 (which is lysosome marker) in Ziyu II-treated CRC cells. In **Figures 4B, C**, CRC cells treated with Ziyu II showed obvious colocalization of LC3 and LAMP1, indicating that Ziyu II improves the merging of the autophagosome with the lysosome. The fusion of autophagy and lysosome was further confirmed in Ziyu II-treated CRC cells, and used a tandem monomeric mRFP-GFP tagged LC3 construct, and noticed that the formation of red fluorescence autolysosomes(GFP-RFP+ signal) increased (**Figures 4D–F**). Together, these findings suggest that Ziyu II treatment induces complete autophagic flux in CRC cells.

### Ziyuglycoside II Induces Autophagy *via* Inhibiting Akt/mTOR Pathway

**Figure 5:**
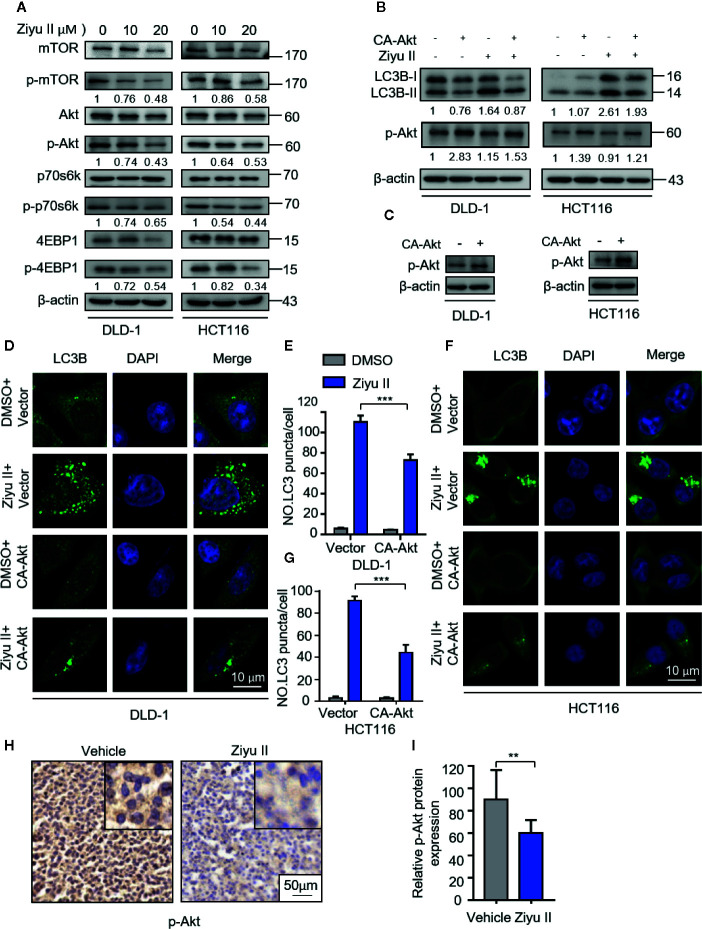
Ziyuglycoside II induces autophagy by inhibiting the Akt/mTOR pathway in colorectal cancer cells. **(A)**. Immunoblotting of phosphorylation of Akt (S473), mTOR (S2448), p70S6K (S424/T421), and 4EBP1 (S65/T70) in cells indicated with the designed concentrations of Ziyu II for 24 h. **(B)**. The empty vector (pECE) or with a constitutively active CA-Akt transfected into the DLD-1 and HCT116 cells for 48 h and then indicated with Ziyu II for another 24 h. The Western blot detected the Akt phosphorylation and LC3 lipidation **(C)**. The formation of endogenous LC3 puncta was evaluated in cells treated as in **(D, F)**, the total number of endogenous LC3 puncta per cell **(E, G)**. Scale bars, 10 μm. All data are means ± SD. ***p < 0.001. Akt expression in orthotopic xenografts was examined by IHC. The charecteristic images were provided as shown **(H)**, and relative immunohistochemical scores were shown **(I)**. Scale bar, 50 μm. ***p < 0.001.

Akt/mTOR is an important signaling pathway for cell survival, which is closely associated with cancer progression and development (Soleimani et al., 2018). It has been previously reported that Ziyu II treatment decreased the phosphorylation level of Akt in human umbilical vein endothelial cells (Nam, et al., 2017). Therefore, we aimed to test whether Akt/mTOR signaling was involved in Ziyu II-induced autophagy in CRC cells. **Figure 5A**, the treatment of the Ziyu II significantly repressed Akt/mTOR signaling pathway, which is evidenced by decreased phosphorylation levels of Akt, mTOR, p70S6K, and 4E-BP1. To further confirm the above results, A transfected a constitutively active form of Akt to rescue Ziyu II-induced Akt/mTOR inhibition. As expected, Akt reactivation notably reduced Ziyu II-induced LC3-II turnover and LC3 puncta accumulation (**Figures 5B–G)**, showing an essential key of the Akt/mTOR signaling pathway in Ziyu II-induced autophagy. Moreover, most of Ziyu II-treated xenografts displayed reduced p-Akt staining (**Figures 5H, I**). Taken together, these findings suggest that Ziyu II induces autophagy by inhibiting Akt/mTOR signaling pathway in CRC cells.

### The Antitumor Activity of Ziyuglycoside II Involves the Autophagy in Colorectal Cancer Cells

**Figure 6:**
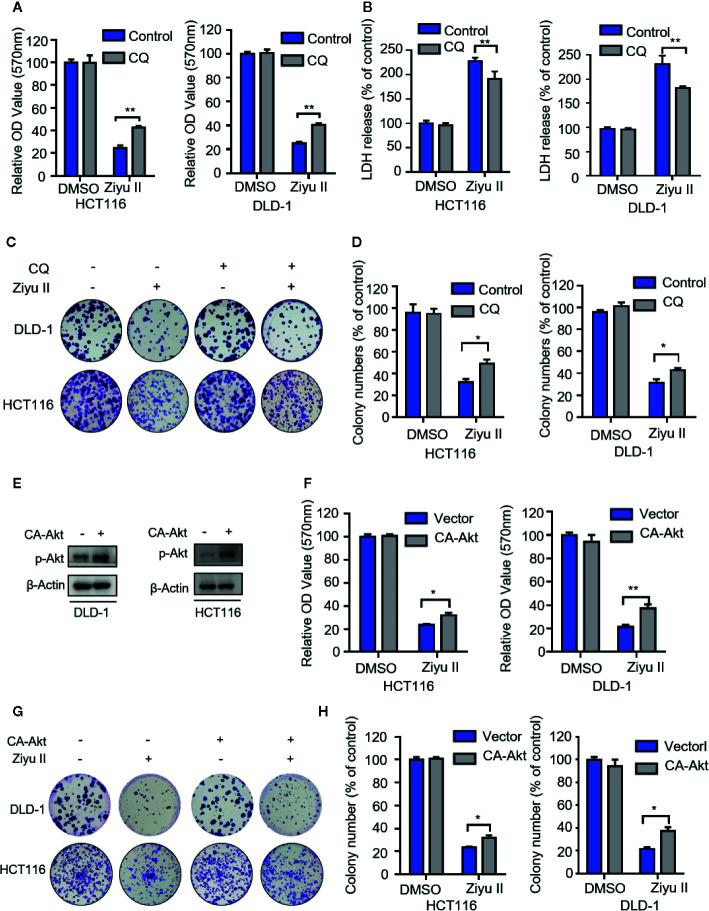
The antiproliferation of the Ziyuglycoside II is mediated through the inhibition of autophagy in the colorectal cancer cells. **(A)**. MTT assay of DLD-1 and HCT116 cells were indicated with or without CQ in the presence or absence of Ziyu II. The characteristic images **(B)** and the quantification of colonies **(C)** were shown. **(D)** LDH release assay in cells treated as in **(A)**. **(E)** The Western blot detected the Akt phosphorylation. **(F)** After transfecting an empty vector (pECE) or with a constitutively active CA-Akt for 48 h, DLD-1 and HCT116 cells were used for MTT analysis and the colony formation assay in indicated with Ziyu II. Characteristic images **(G)** and quantification of colonies **(H)** were shown. All data are means ± SD. *p < 0.05 **p < 0.01.

Next intend to evaluate whether autophagy was involved in the anti-CRC effect of Ziyu II. First, Ziyu II combined with CQ or 3-mA was used to treat colorectal cancer cells. As **Figure 6A** and **Figure S4A** show, combinational treatment of CQ, or 3-MA with Ziyu II partially restored Ziyu II-induced growth inhibition in CRC cells. Moreover, LDH release assay also unveiled that CQ or 3-MA offset Ziyu II-induced cytotoxicity (**Figure 6B** and **Figure S4B**). A comparable increase in cell proliferation was also perceived in Ziyu II-treated CRC cells in mixture with CQ or 3-MA, as proved by colony formation analysis (**Figures 6C, D** **and** **Figures S4C, D**). Furthermore, resemble results were collected by inhibition of autophagy by transfecting a constitutively active form of Akt (**Figures 6E–H** **and** **Figures S4E, F**). Collectively, these data demonstrate that autophagy is involved in the growth inhibition of colorectal cancer induced by Ziyu II.

Further investigated whether Ziyu II enhanced the sensitivity of 5-fluorouracil (5-FU), which is the first-line chemotherapy drug for colorectal cancer. As indicated, Compared with Ziyu II-treatment, Ziyu II combined with 5-FU significantly repress the growth (**Figure S5A**) and proliferation rate (**Figures S5B, C**) of colorectal cancer cells, indicating that Ziyu II can effectively make colorectal cancer cells sensitive to 5-FU treatment.

## Discussion

Ziyu II is one of the significant active components of *S. officinalis L*. The previous investigations have demonstrated the anticancer effect of Ziyu II in a variety of cancer types. In this research, we demonstrated its anticancer effect against CRC cells both *in vitro* and *in vivo*. We showed that ROS-induced apoptosis and the autophagy induced by the inhibition of Akt/mTOR signaling both contribute to the growth inhibition of Ziyu II against CRC cells. Also, Ziyu II acts synergistically with the first-line chemotherapeutic drugs 5-fluorouracil in the suppression of CRC cell growth, suggesting Ziyu II as a potential anticancer drug or chemosensitizer for CRC treatment.

One of the key points in the development of anti-cancer drugs is to find active ingredients from natural substances. At present, according to a large number of investigations, TCM is a kind of multi-target anti-cancer drug by inhibiting cell proliferation, causing cell cycle arrest, promoting cell apoptosis, inhibiting neovascularization, and blocking invasion and metastasis of tumors (Liu et al., 2019; Xiang et al., 2019). *S. officinalis* L., a commonly used Chinese herbal medicine, used in the treatment of inflammation, metabolic diseases, and a variety of cancers. Ziyu II is one of the important active components of *S. officinalis L*. Up to the present, the anticancer effects of Ziyu II have been reported in human breast cancer and gastric carcinoma, and the concrete underlying mechanisms are complicated. In diverse cancer cells, Ziyu II has complex antitumor effects. For example, in MCF-7 and MDA-MB-231 human breast cells (Zhu X. et al., 2013; Zhu et al., 2014), it shows G2/M arrest and significant apoptosis, while in BGC-823 human gastric carcinoma cells (Zhu A. K. et al., 2013), it shows the anti-angiogenesis effect. Hence, this indicates that the effect of Ziyu II on cancer cells varies with cell types. Therefore, to clarify this point, we investigated the anticancer effect and mechanism of Ziyu II on colorectal cancer cells. In this study, the molecular mechanism of the anti-cancer effect of Ziyu II in CRC cells is inducing both apoptosis and autophagy.

The reutilization of the drug has been considered to be an attractive strategy, which can provide more effective choices for cancer patients, and has significant advantages over the *de novo* drug development, such as cheaper, faster and safer (Pushpakom et al., 2019). Previous studies have demonstrated that many types of TCM have been identified as potential anticancer drugs *via* drug repurposing screen. For example, Shikonin is a kind of medicinal compound extracted from Lithospermum erythrorhizon. herbal medicine, which has been used to treat burns, cuts, and injuries caused by diseases for centuries (Zhang et al., 2018). Growing evidence has demonstrated Shikonin as a potential anticancer drug or chemosensitizer *via* inducing apoptosis and necroptosis (Jeung et al., 2016; Kim et al., 2017). Our previous works have also identified several natural compounds that exhibit potential anticancer activities (Wang et al., 2011; Huang et al., 2017). Among these candidates, Ziyu II, a major active component of *S. officinalis L* used for anti-inflammation and anti-oxidation, has been found to suppress CRC growth by apoptosis and autophagy in the present research, further highlighting the important role of drug repurposing in cancer drug development.

Apoptosis and autophagy are involved in the physiological process of cells such as growth, differentiation, death, and also closely related to the development of tumors (Hussey et al., 2008; Sun et al., 2018). The machinery of apoptosis and autophagy are quite complex, with many signaling pathways involved. Apoptosis can be triggered by multiple factors including ROS (Diwanji and Bergmann, 2018). Meanwhile, CRC is characterized by partially inhibited apoptosis, which in turn provides a selective advantage for the survival of tumors and becomes a major obstacle to treatment (Yang et al., 2009). As for the autophagy, a wealth of research suggests that cancer therapy have identified autophagy activation induced by chemotherapy or radiotherapy, however, the role of autophagy is different in cancer progression (White and DiPaola, 2009). In general, autophagy is considered to be a pro-survival mechanism of cancer cells by removing damaged or necrotic cells and recovering nutrients (Fulda and Kogel, 2015). Recently the significant finding is that autophagy induced by certain chemotherapeutic drugs may have inhibitory effect on cancer cells, revealing two additional functional forms of autophagy, one of which is cytotoxic function leading to autophagy cell death or promoting cell apoptosis, and the other is cell inhibition function, which may inhibit cell proliferation in a non-apoptotic manner (Kubisch et al., 2013). In this study, we reviewed the anti-cancer effect of Ziyu II in CRC cells both *in vitro* and *in vivo*. Interestingly, we noticed that Ziyu II-induced apoptosis and autophagy through the different pathways in CRC cells. Indeed, Ziyu II has been reported to induce apoptosis in which ROS plays important roles in several cancer types. Hence, our present study is mainly focused on exploring whether there exist other possibilities besides apoptosis in the anti-tumor effect of Ziyu II. Our results finally demonstrated that Ziyu II also exhibited the anti-tumor effect against CRC cells by inducing autophagic cell death besides the apoptosis. Further study found that Ziyu II-induced autophagy through inhibition of the Akt/mTOR signaling pathway which has been evidenced by attenuated phosphorylation of Akt, mTOR, and downstream substrates. Overall, our findings suggest that targeting the Akt/mTOR signaling pathway may deserve exploration as a potential therapeutic strategy for CRC treatment and imply that Ziyu-II might serve as a promising agent for autophagy stimulators.

To sum up, our findings indicate that the Chinese herbal medicine extract-Ziyu II inhibits colorectal cancer growth by orchestrating Akt/mTOR-mediated autophagic cell death and ROS-induced apoptosis. These findings provide new insights into the mechanisms of Ziyu II-induced colorectal cancer suppression and provide the basis for the rational application of Ziyu II for the treatment of colorectal cancer.

## Author Contributions

Conceived and designed the experiments: CB and YT. Performed the experiments: CB, ZZ, and YC. Analyzed the data: CB, H-YZ, and YT. Wrote the manuscript: CB, LZ, and YT.

## Conflict of Interest

The authors declare that the research was conducted in the absence of any commercial or financial relationships that could be construed as a potential conflict of interest.

## Supplementary Material

Supplementary Figure 1 Ziyu II has no obvious toxicity in mice. Hematoxylin-eosin (H&E) staining of Lung, liver, kidney, heart and spleen from nude mice treated vehicle or Ziyu II. Scale bar, 50 μm.

Supplementary Figure 2 Ziyuglycoside II induces apoptosis in colorectal cancer cells both *in vitro* and *in vivo.* Immunoblotting of Cleaved-PARP in cells indicated with the designated concentrations of Ziyu II for 24 h **(A)**. Immunoblotting of Cleaved-PARP in cells indicated with ZVAD in the presence or absence of Ziyu II (20 uM) for 24 h **(B)**. Immunohistochemical staining of cleaved-caspase 3 (CC3) **(C)** in tumors from vehicle or Ziyu II-treated mice bearing DLD-1 subcutaneous tumor xenografts. Relative immunohistochemical scores were shown **(D)**.Scale bar, 50 μm. ***, p< 0.001.

Supplementary Figure 3 The formation of LC3 in colorectal cancer cells by shRNA-mediated silencing of Atg5 gene. **(A)**. The cells were transfected with empty vector or siAtg5 for 48 h and then indicated with Ziyu II (20 μ m) for 24 h. Atg5 was detected by immunoblotting and **(B, D)** the formation of endogenous LC3 puncta was assessed in cells indicated as in A and **(C, E)** total number of endogenous LC3 puncta per cell. Scale bars: 10 μm. All data are means ± SD. ***, *p* < 0.001.

Supplementary Figure 4 The inhibition of autophagy represses the antiproliferative effect of Ziyu II in colorectal cancer cells. Colony formation assay of DLD-1 and HCT116 cells were treated with or without 3-MA in the presence or absence of Ziyu II. The chracteristic images **(A)** and the count of colonies **(C)** were shown. MTT assay **(B)** of ells were treated with or without 3-MA in the presence or absence of Ziyu II. LDH release assay in cells treated as in **(B)**. **(F)**. the constitutively active CA-Akt or the empty vector transfected the colorectal cancer cells for 48 h, and then treated with Ziyu II. the cytotoxicity was detected by the release of LDH. All data are means ± SD. *, *p* < 0.05; **, *p* < 0.01.

Supplementary Figure 5 Ziyu II enhances the anti-cancer efficacy of 5-Fluorouracil in colorectal cancer cells. **(A)**. Effect of Ziyu II combined with 5-FU on the growth of colorectal cancer cells (**B**). Colony formation assay of CRC cells indicated with 15 μM Ziyu II in the presence or absence of 10 μM 5-FU. The characteristic images **(B)** and the count of colonies **(C)** were shown. ***, *p* < 0.001.

## Abbreviations

CRC, Colorectal cancer; Ziyu II, 3β-3-α-L-arabinopyranosyloxy-19-hydroxyurs-12-en-28-oic acid; ROS, reactive oxygen species; CMA, chaperone-mediated autophagy; TCM, traditional Chinese medicine; β-actin, actin, beta; ATG5, autophagy related 5; ATG7, autophagy related 7; MAP1LC3B/LC3B, microtubule associated protein 1 light chain 3 beta; 3-MA, 3-methyladenine; 5-FU:5-fluorouracil; CQ, chloroquine; DMSO, dimethyl sulfoxside; EdU, 5-ethynyl-20-deoxyuridine; LDH, lactate dehydrogenase; MTT, 3-(4,5-dimethylthiazol-2-yl)-2,5-diphenyltetrazolium bromide.
