# Supplementary figures and images for: Repurposing Ziyuglycoside II Against Colorectal Cancer via Orchestrating Apoptosis and Autophagy
Source: Front Pharmacol. 2020 Sep 18;11:576547. doi: 10.3389/fphar.2020.576547 (PMC7533566; doi:10.3389/fphar.2020.576547)

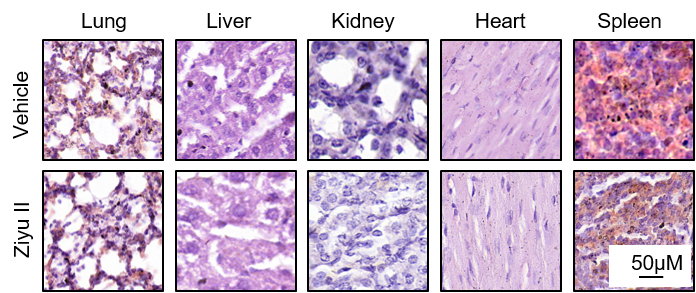

Supplement: Supplementary Figure 1 — Ziyu II has no obvious toxicity in mice. Hematoxylin-eosin (H&E) staining of Lung, liver, kidney, heart and spleen from nude mice treated vehicle or Ziyu II. Scale bar, 50 μm. [file Image_1.tif]

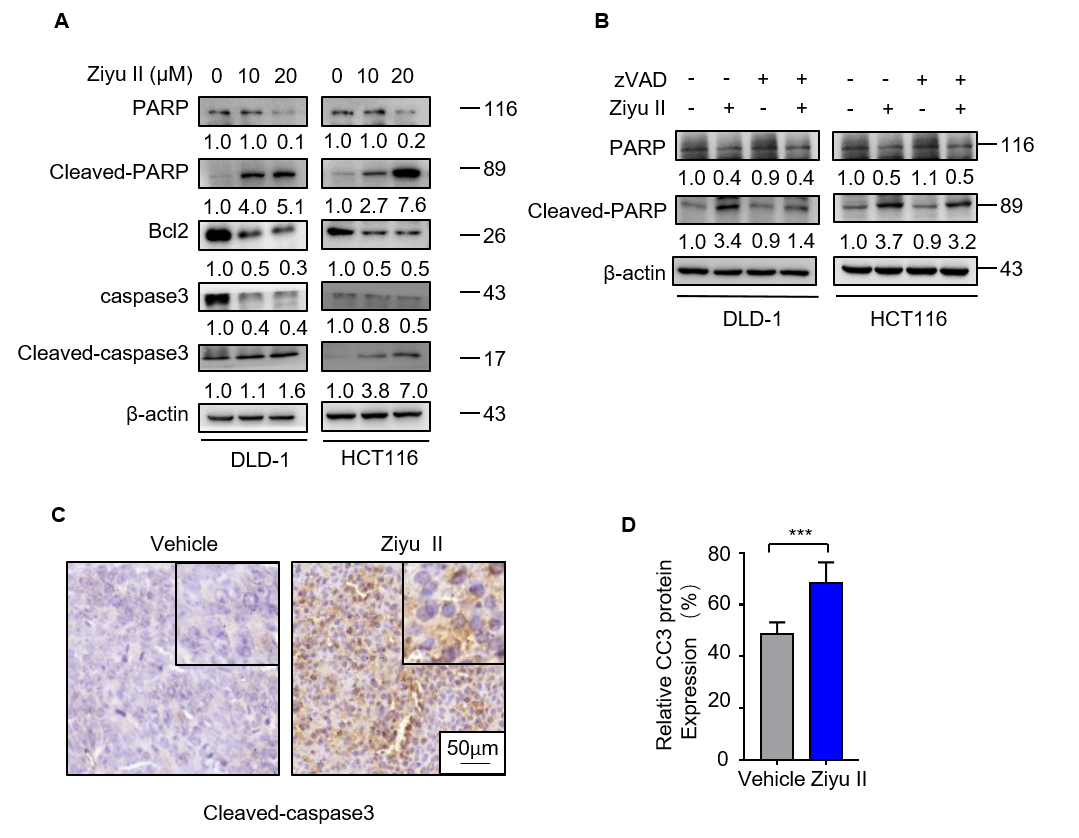

Supplement: Supplementary Figure 2 — Ziyuglycoside II induces apoptosis in colorectal cancer cells both in vitro and in vivo. Immunoblotting of Cleaved-PARP in cells indicated with the designated concentrations of Ziyu II for 24 h (A). Immunoblotting of Cleaved-PARP in cells indicated with ZVAD in the presence or absence of Ziyu II (20 uM) for 24 h (B). Immunohistochemical staining of cleaved-caspase 3 (CC3) (C) in tumors from vehicle or Ziyu II-treated mice bearing DLD-1 subcutaneous tumor xenografts. Relative immunohistochemical scores were shown (D).Scale bar, 50 μm. ***, p< 0.001. [file Image_2.tif]

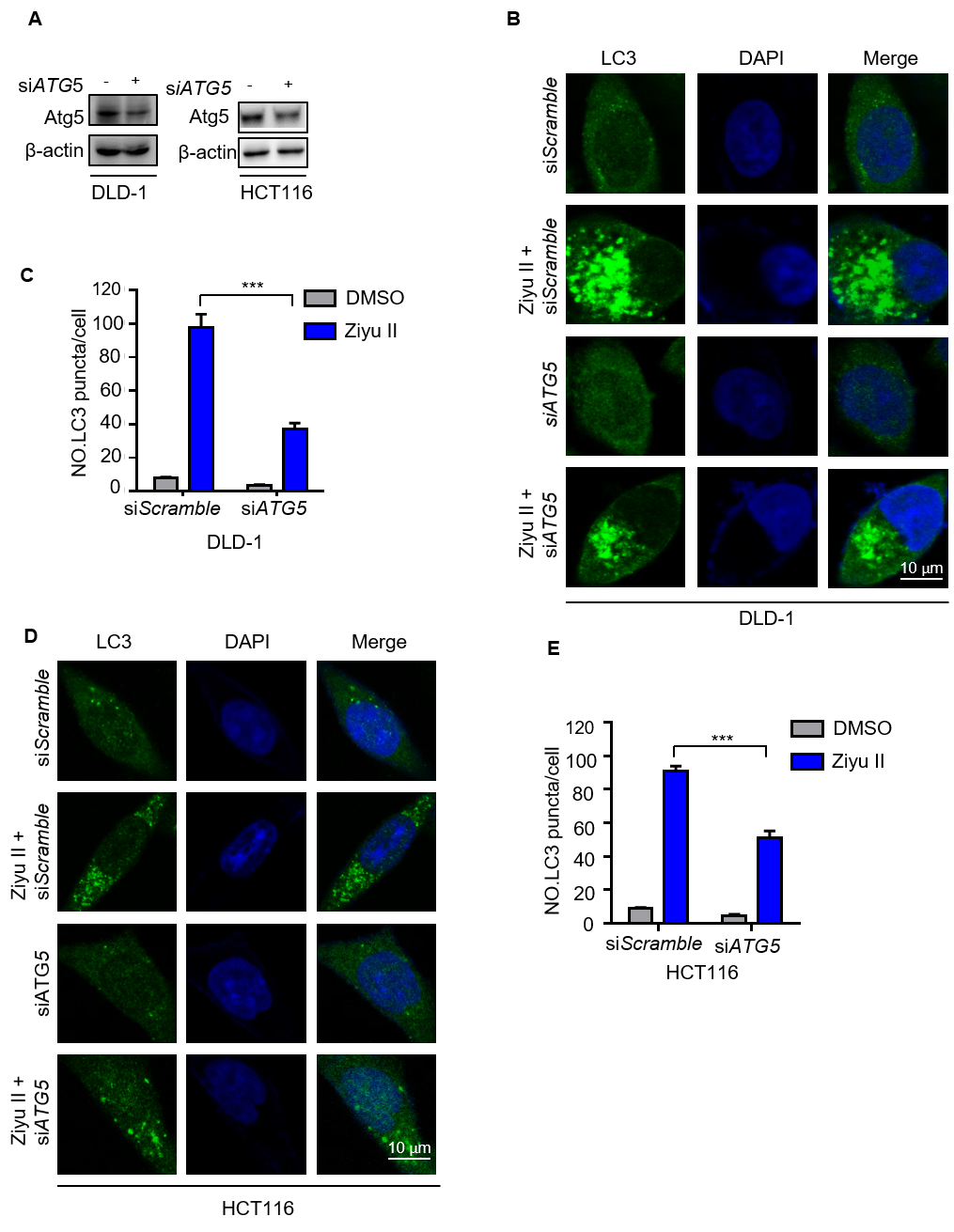

Supplement: Supplementary Figure 3 — The formation of LC3 in colorectal cancer cells by shRNA-mediated silencing of Atg5 gene. (A). The cells were transfected with empty vector or siAtg5 for 48 h and then indicated with Ziyu II (20 μ m) for 24 h. Atg5 was detected by immunoblotting and (B, D) the formation of endogenous LC3 puncta was assessed in cells indicated as in A and (C, E) total number of endogenous LC3 puncta per cell. Scale bars: 10 μm. All data are means ± SD. ***, p < 0.001. [file Image_3.tif]

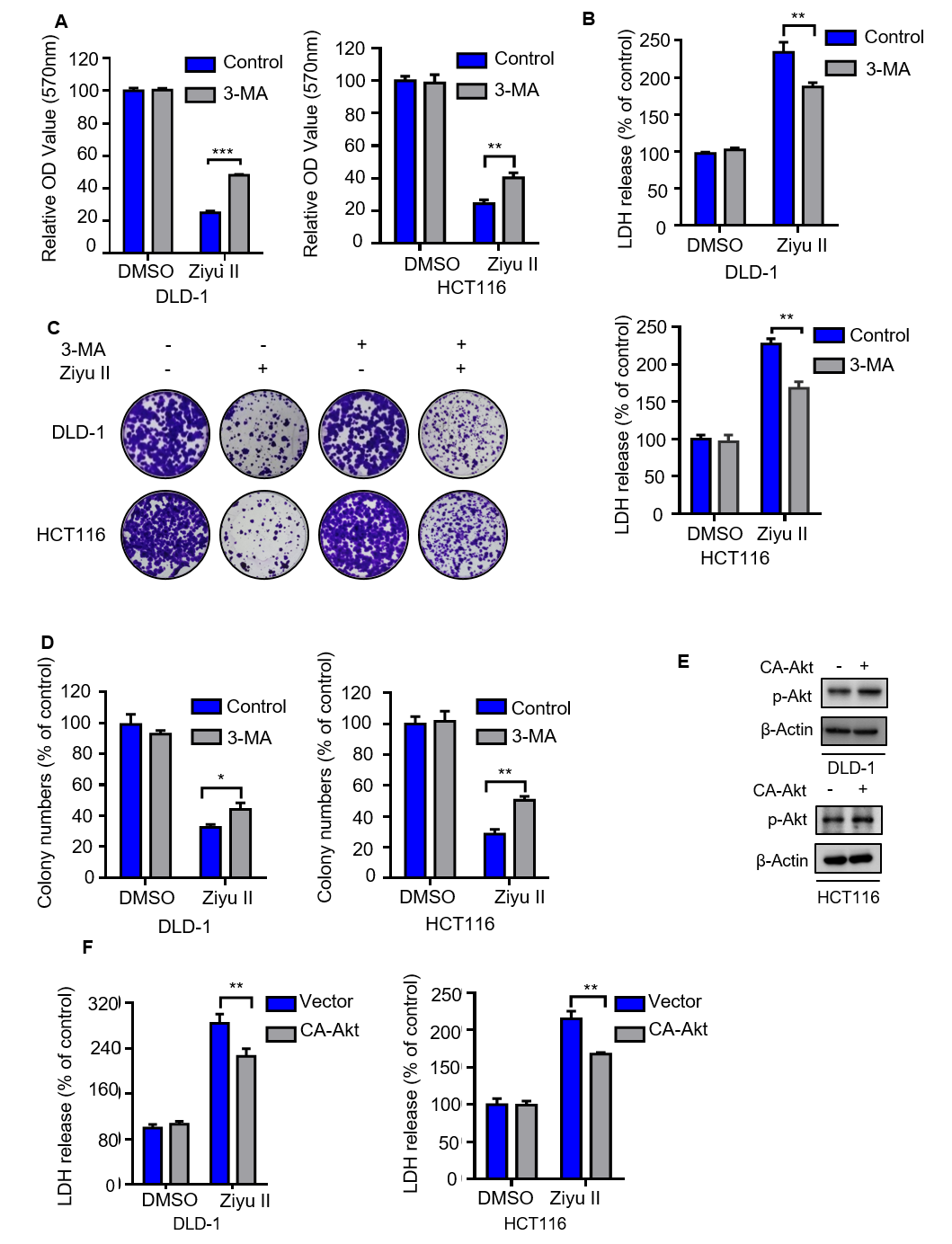

Supplement: Supplementary Figure 4 — The inhibition of autophagy represses the antiproliferative effect of Ziyu II in colorectal cancer cells. Colony formation assay of DLD-1 and HCT116 cells were treated with or without 3-MA in the presence or absence of Ziyu II. The chracteristic images (A) and the count of colonies (C) were shown. MTT assay (B) of ells were treated with or without 3-MA in the presence or absence of Ziyu II. LDH release assay in cells treated as in (B). (F). the constitutively active CA-Akt or the empty vector transfected the colorectal cancer cells for 48 h, and then treated with Ziyu II. the cytotoxicity was detected by the release of LDH. All data are means ± SD. *, p < 0.05; **, p < 0.01. [file Image_4.tif]

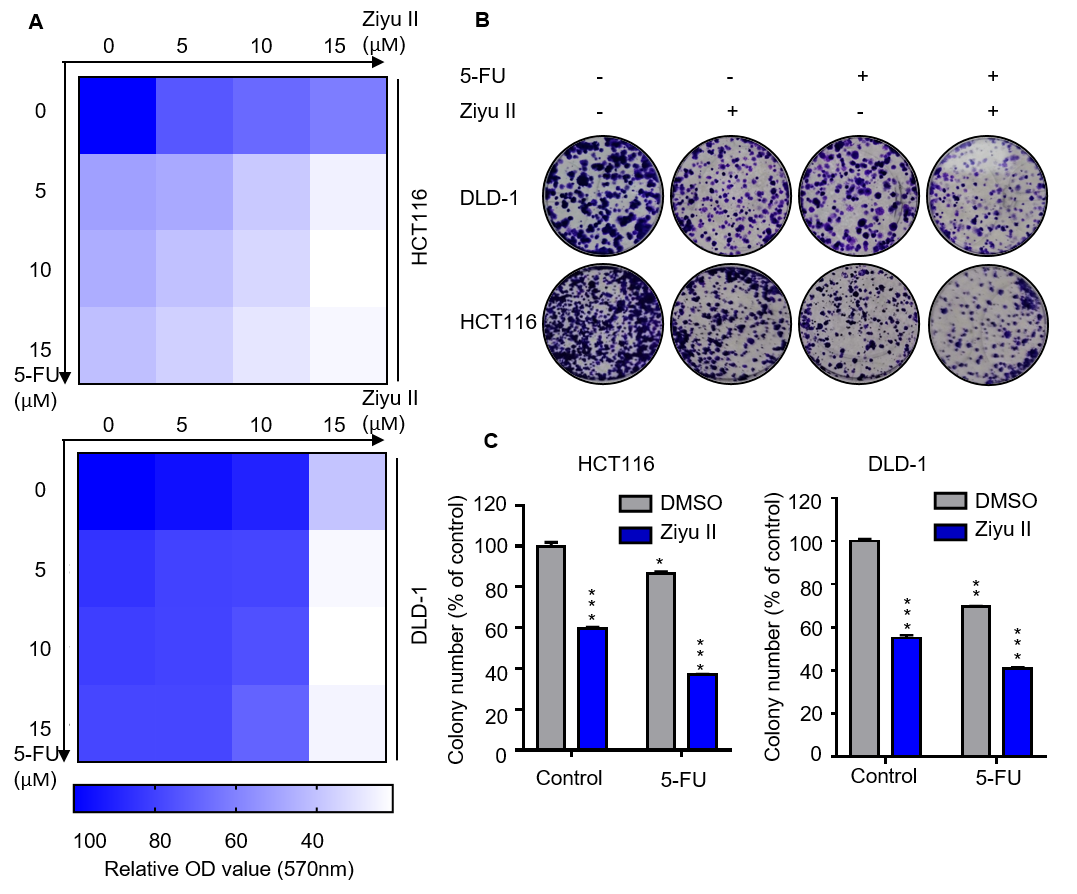

Supplement: Supplementary Figure 5 — Ziyu II enhances the anti-cancer efficacy of 5-Fluorouracil in colorectal cancer cells. (A). Effect of Ziyu II combined with 5-FU on the growth of colorectal cancer cells (B). Colony formation assay of CRC cells indicated with 15 μM Ziyu II in the presence or absence of 10 μM 5-FU. The characteristic images (B) and the count of colonies (C) were shown. ***, p < 0.001. [file Image_5.tif]
